# Five New Taraxerene-Type Triterpenes from the Branch Barks of *Davidia involucrata*

**DOI:** 10.3390/molecules191117619

**Published:** 2014-10-30

**Authors:** Qing-Wei Tan, Ming-An Ouyang, Qi-Jian Chen, Zu-Jian Wu

**Affiliations:** 1 Key Laboratory of Plant Virology of Fujian Province, Institute of Plant Virology, Fujian Agriculture and Forestry University, Fuzhou 350002, Fujian, China; E-Mails: tanqingwei@fafu.edu.cn (Q.-W.T.); fafuchenqijian@163.com (Q.-J.C.); wuzujian@126.com (Z.-J.W.); 2 Key Laboratory of Bio-Pesticide and Chemistry-Biology, Ministry of Education, Fujian Agriculture and Forestry University, Fuzhou 350002, Fujian, China

**Keywords:** *Davidia involucrata*, Nyssaceae, taraxerene, triterpenoid

## Abstract

Five new taraxerene-type triterpenes, 2-nor-D-friedoolean-14-en-28-ol (**1**), 2-nor-d-friedoolean-14-en-3α,28-diol (**2**), 6α-hydroxy-2-nor-D-friedoolean-14-en-3,21-dione (**3**), 6α,11α,29-trihydroxy-D-friedoolean-14-en-3,16,21-trione (**4**), and 6α,23,29-trihydroxy-D-friedoolean-14-en-3,16,21-trione (**5**), were isolated from the MeOH extract of the branch barks of *Davidia involucrata*, together with five known compounds. Their structures were elucidated by means of various spectroscopic analyses. Five of the identified compounds showed moderate cytotoxicities against the cell proliferation of SGC-7901, MCF-7, and BEL-7404.

## 1. Introduction

*Davidia involucrata* Baill., an ornamental tree known as the Chinese dove tree or handkerchief tree, is the only species in genus *Davidia*. *D. involucrata* is a relic deciduous tree species of the Tertiary period with important ecological, scientific and horticultural values [1,2,3]. An initial and the only report of study on chemical components of *D. involucrata* besides our program appeared in 1989, which revealed the presence of sterols, tannins and triterpenes [4]. The branch barks of *D. involucrata* have been intensively studied in our previous work and were found containing diverse constituents including flavonoids, alkaloids, lignans, and phenols, among which were three alkaloids including vicosamide, strictosidinic acid and puimiloside, which were proposed to be the intermediate precursors between strictosamide and camptothecin [5,6,7,8,9,10,11,12]. Moreover, we have recently reported the identification of two novel 2-nor-ursane triterpenes having a unique five-membered A-ring from the water insoluble fraction of the branch barks of *D. involucrata* [13].

**Figure 1:**
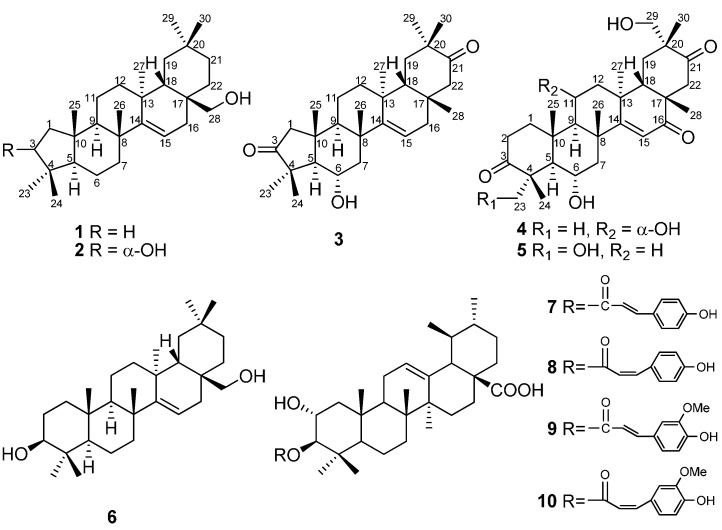
Chemical structures of compounds isolated from the branch barks of *Davidia involucrata*.

Our continuing work led to the isolation of another five novel triterpenoids, namely davinvolunols A-B (**1**–**2**) and davinvolunones A-C (**3**–**5**), together with a known taraxerene triterpene, myricadiol (**6**) [14,15,16], and four known triterpene esters, 3β-*O*-*trans*-*p*-coumaroyl-2α-hydroxy-urs-12-en-28-oic acid (**7**) [17,18,19], 3β-*O*-*cis*-*p*-coumaroyl-2α-hydroxy-urs-12-en-28-oic acid (**8**), 3β-*O*-*trans*-*p*-feruloyl-2α-hydroxy-urs-12-en-28-oic acid (**9**), 3β-*O*-*cis*-*p*-feruloyl-2α-hydroxy-urs-12-en-28-oic acid (**10**) [19] (Figure 1). Compounds **1**–**3** feature a contracted five-membered A-ring, whereas **4**–**5** are highly oxidized. The novel structures were unequivocally determined by extensive spectroscopic analysis and comparison with literature data. We herein report in this paper the structure elucidation of the new triterpenes, as well as the *in vitro* cytotoxic activities against three tumor cell lines (SGC-7901, MCF-7 and BEL-7404) of the isolated compounds.

## 2. Results and Discussion

**Figure 2:**
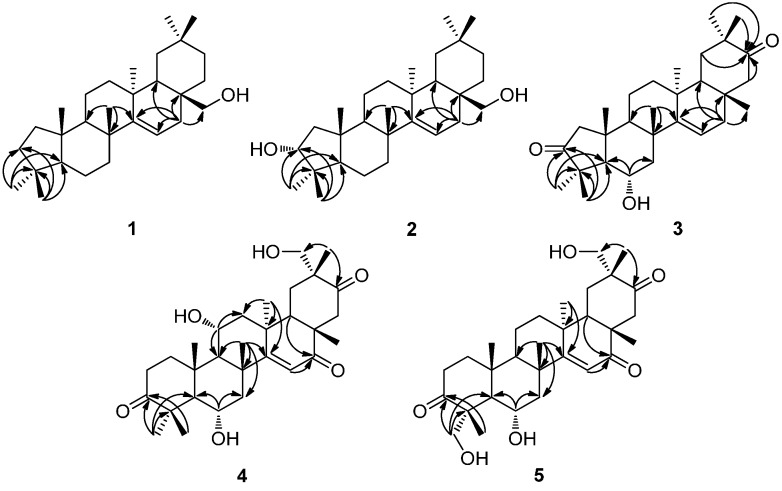
Key HMBC (H→C) correlations of compounds **1**–**5**.

**Figure 3:**
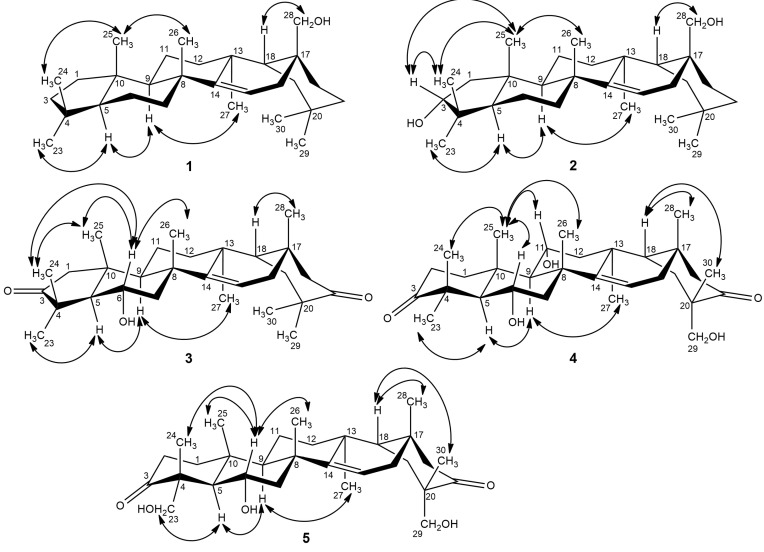
Key ROESY (H↔C) correlations of compounds **1**–**5**.

Compound **1**, a white amorphous powder, had a molecular formula of C_29_H_48_O as determined by HR-TOF-MS at *m/z* = 435.3596 [M + Na]^+^ (calcd for C_29_H_48_ONa, 435.3603). The IR spectrum exhibited absorption bands due to hydroxyl (3560 cm^−1^) and olefinic (1640 cm^−1^) groups. Observed in the ^1^H-NMR (500 MHz, CDCl_3_ and CD_3_OD) spectrum were signals for seven tertiary methyl groups at δ_H_ 0.87, 0.94, 0.94, 1.05, 1.07, 1.08 and 1.22 (s, each 3H), while resonances at δ_H_ 3.10 and 3.23 (d, *J* = 10.9 Hz, each 1H) were attributed to proton signals attached to an oxygenated methylene carbon. In addition, one olefinic proton at δ_H_ 5.51 (dd, *J* = 8.2, 3.1 Hz, 1H) of a trisubstituted double bond, coupled with the protons of a methylene at δ_H_ 1.68 (dd, *J* = 15.2, 3.1 Hz, 1H) and 2.13 (dd, *J* = 15.2, 8.2 Hz, 1H) as deduced from their coupling constants, was recognized in the ^1^H-NMR spectrum. The ^13^C-NMR (125 MHz, CDCl_3_ and CD_3_OD) spectrum of **1** showed 29 carbon signals, which were classified from DEPT and HSQC data as seven methyls, ten methylenes, three methines, six quaternary carbons, one oxygen-bearing secondary carbon and a trisubstituted double bond. One of the six degrees of unsaturation came from a trisubstituted double bond at δ_C_ 116.0 and 158.5, and the remaining five degrees of unsaturation were therefore indicative of a pentacyclic skeleton. The characteristics of NMR data of compound **1** were comparable to those of myricadiol (**6**) [13]. Comparison of the MS, 1D- and 2D-NMR data of **1** with those of **6** revealed that they both shared the same B/C/D/E rings, indicating that **1** might bear a contracted five-membered A-ring. This inference was confirmed by the HMBC and ROESY experiments. The key HMBC correlations (Figure 2) from H_3_-23 (δ_H_ 1.22, s, 3H) and H_3_-24 (δ_H_ 0.94, s, 3H) to C-3 (δ_C_ 29.8), C-4 (δ_C_ 47.5) and C-5 (δ_C_ 55.7) enabled the establishment of an unusual five-membered A-ring. The observed ROESY cross-peaks (Figure 3) of H_3_-24/H_3_-25, H_3_-25/H-26, and H-18/H-28 indicated that they were on the same side of the molecule, and were arbitrarily assigned as β-oriented. As a consequence, the ROESY correlations of H_3_-23/H-5, H-5/H-9, and H-9/H_3_-27 revealed that they were α-configured. Hence, the structure of compound **1** was determined to be 2-nor-d-friedoolean-14-en-28-ol, and this substance has been accorded the trivial name davinvolunol A.

Compound **2** was isolated as a white amorphous solid. It was assigned to have a molecular formula of C_29_H_48_O_2_ by HR-TOF-MS at *m/z* = 451.3546 [M + Na]^+^ (calcd for C_29_H_48_O_2_Na, 451.3552). The ^1^H and ^13^C-NMR spectra of **2** were highly similar to those of **1** except for the absence of methylene proton signals at δ_H_ 2.01 and 2.25 (m, each 1H) with the presence of an additional oxymethine proton signal at δ_H_ 3.18 (dd, *J* = 9.8, 6.8 Hz, 1H) instead, suggesting that a hydroxyl group was attached to C-3. This was confirmed by the key HMBC correlations (Figure 2) from H_3_-23 (δ_H_ 1.05, s, 3H) and H_3_-24 (δ_H_ 0.91, s, 3H) to C-3 (δ_C_ 77.3). The ROESY correlations (Figure 3) of H-3/H_3_-24, H_3_-25 implied that H-3 was α-orientated, and thus 3-OH was in β-orientation. Other observed ROESY effects indicated the relative configuration of the remaining part of the molecule of **2** was identical with that of **1**. Thus, the structure of compound **2** (davinvolunol B) was elucidated as 2-nor-d-friedoolean-14-en-3α,28-diol.

Compound **3**, a white amorphous solid, presented a molecular formula of C_29_H_44_O_3_, as determined by HR-TOF-MS at *m/z* = 463.3180 [M + Na]^+^ (calcd for C_29_H_44_O_3_Na, 463.3188) with eight double-bond equivalents. The IR spectrum revealed the presence of hydroxyl (3437 cm^−1^), carbonyl (1726 cm^−1^) and olefinic (1635 cm^−1^) groups. The ^1^H-NMR (400 MHz, CDCl_3_) spectrum showed eight tertiary methyls at δ_H_ 0.78, 0.82, 1.02, 1.05, 1.06, 1.11, 1.29 and 1.32 (s, each 3H), and a characteristic olefinic proton signal at δ_H_ 5.60 (dd, *J* = 8.1, 2.9 Hz, 1H) coupled with the protons of a methylene at δ_H_ 1.79 (dd, *J* = 14.5, 8.1 Hz, 1H) and 2.05 (dd, *J* = 14.5, 2.9 Hz, 1H), as deduced from their coupling constants, indicative of the same D-friedoolean-14-en skeleton of **3** as compounds **1** and **2**. In addition, an oxygenated methylene at δ_H_ 3.91 (ddd, *J* = 15.0, 10.9, 4.1 Hz, 1H) coupled with the protons of a methylene at δ_H_ 1.38 (t, *J* = 10.9 Hz, 1H) and 2.28 (dd, *J* = 10.9, 4.1 Hz, 1H) was observed in the ^1^H-NMR spectrum of **3**. The ^13^C-NMR (100 MHz, CDCl_3_) spectrum, along with HSQC and DEPT data, resolved 29 carbons that came from eight methyls, seven methylenes, three methines, six quaternary carbons, an oxygenated secondary carbon, two keto carbonyls and a trisubstituted double bond. The HMBC correlations (Figure 2) from H_3_-23 (δ_H_ 1.29, s, 3H) and H_3_-24 (δ_H_ 1.32, s, 3H) to C-3 (δ_C_ 219.7) rationalized the existence of a keto group at C-3, while another keto group was assigned to C-21 as deduced from the HMBC correlations from H_3_-29 (δ_H_ 1.06, s, 3H) and H_3_-30 (δ_H_ 1.05, s, 3H) to C-21 (δ_C_ 219.7). An OH group was attached at C-6 as deduced from the key HMBC correlations of H-6 (δ_H_ 3.91, ddd, *J* = 15.0, 10.9, 4.1 Hz, 1H) to C-5 (δ_C_ 58.8) and C-7 (δ_C_ 50.4), as well as the observation of an AMX spin system of H-5 (δ_H_ 1.61, d, *J* = 10.9 Hz, 1H), H-6 (δ_H_ 3.91, ddd, *J* = 15.0, 10.9, 4.1 Hz, 1H), H-7a (δ_H_ 1.38, t, *J* = 10.9 Hz, 1H) and H-7b (δ_H_ 2.28, dd, *J* = 10.9, 4.1 Hz, 1H) in the ^1^H-NMR spectrum. A Δ^14^ double bond was determined by the HMBC correlations from H_3_-26 (δ_H_ 1.11, s, 3H) to C-14 (δ_C_ 157.3), and from H-16a (δ_H_ 1.79, dd, *J* = 14.5, 8.1 Hz) and H-16b (δ_H_ 2.05, dd, *J* = 14.5, 2.9 Hz, 1H) to C-14 (δ_C_ 157.3) and C-15 (δ_C_ 116.5). The relative stereochemistry of **3** was established by the ROESY spectrum. The ROESY correlations (Figure 3) of H-6/H_3_-24, H_3_-25, H_3_-26 and H-18/H_3_-28, H_3_-30 implied that H-6, H_3_-24, H_3_-25, H_3_-26, H_3_-28, H_3_-30 were cofacial and placed in β-orientation, and thus indicated the α-orientation of the hydroxyl group at C-6. The structure of compound **3** was therefore determined as 6α-hydroxy-2-nor-d-friedoolean-14-en-3,21-dione and named davinvolunone A.

Compound **4** was obtained as a white amorphous powder. Its molecular formula was deduced to be C_30_H_44_O_6_ by the HR-TOF-MS at *m/z* 523.3030 [M + Na]^+^ (calcd for C_30_H_44_O_6_Na, 523.3036). The IR spectrum exhibited the presence of hydroxyl (3500 cm^−1^), carbonyl (1700 cm^−1^) and conjugated carbonyl (1610 cm^−1^) functional groups. The ^1^H-NMR spectrum of compound **4** showed the presence of proton signals for seven tertiary methyls at δ_H_ 0.98, 1.06, 1.07, 1.22, 1.26, 1.35 and 1.36 (s, each 3H), and resonances at δ_H_ 3.98 (ddd, *J* = 15.2, 11.1, 4.2 Hz, 1H) and δ_H_ 4.28 (t, *J* = 7.0 Hz, 1H) attributed to proton attached to two oxygenated methine carbons, as well as signals of two protons at δ_H_ 3.26 and 3.71 (d, *J* = 10.3 Hz, each 1H) attached to an oxygenated methylene carbon. In addition, an olefinic proton signal at δ_H_ 5.96 (br. s, 1H) was observed in the ^1^H-NMR spectrum. The ^13^C-NMR (400 MHz, CD_3_OD) including DEPT experiments showed that four of the nine degrees of unsaturation came from one trisubstituted double bond at δ_C_ 118.8 and 177.9, and three keto carbonyls at δ_C_ 206.3, 219.9 and 222.5. The remaining five degrees of unsaturation were therefore indicative of a pentacyclic skeleton. A Δ^14^ double bond was determined by the key HMBC correlations (Figure 2) from H_3_-26 (δ_H_ 1.26, s, 3H) and H_3_-27 (δ_H_ 1.22, s, 3H) to C-14 (δ_C_ 177.9). Key HMBC correlations from the olefinic proton at δ_H_ 5.96 (br. s, 1H) to a carbonyl at δ_C_ 206.3 indicated that one keto group was placed at C-16. Another two keto groups were assigned to C-3 and C-21, which was confirmed by the observed key HMBC correlations from H_3_-23 (δ_H_ 1.35, s, 3H) and H_3_-24 (δ_H_ 1.36, s, 3H) to C-3 (δ_C_ 222.5), and correlations from H_3_-30 (δ_H_ 1.06, s, 3H) to C-21 (δ_C_ 219.9). Moreover, three hydroxyls were attached to C-6, C-11 and C-29 respectively, which was supported by the key HMBC correlations observed from H-5 (δ_H_ 1.78, d, *J* = 11.1 Hz, 1H), H-7a (δ_H_ 1.49, t, *J* = 11.1 Hz, 1H) and H-7b (δ_H_ 2.30, dd, *J* = 11.1, 4.2 Hz, 1H) to C-6 (δ_C_ 67.5), from H-9 (δ_H_ 1.60, d, *J* = 7.0 Hz, 1H) and H-12a (δ_H_ 1.98, d, *J* = 14.5 Hz, 1H) to C-11 (δ_C_ 65.9), and from H_3_-30 (δ_H_ 1.06, s, 3H) to C-29 (δ_C_ 71.2). ROESY experiment was undertaken to establish the relative configuration of **4**. The significant ROESY correlations (Figure 3) of H-11/H_3_-25, H-6/H_3_-24, H_3_-25 and H_3_-26, H-18/ H_3_-28 and H_3_-30 indicated that they were on the same side of the molecule, and were assigned as β-oriented. As a consequence, the ROESY correlations of H_3_-23/H-5, H-5/H-9 and H-9/H_3_-27 revealed that they were α-configured. Thus, the structure of compound **4 ** (davinvolunone B) was determined as 6α,11α,29-trihydroxy-d-friedoolean-14-en-3,16,21-trione.

Compound **5**, obtained as a white amorphous powder, was assigned to have a molecular formula of C_30_H_44_O_6_ by HR-TOF-MS at *m/z* = 523.3030 [M + Na]^+^ (calcd for C_30_H_44_O_6_Na, 523.3036), which was an isomer of **4**. The NMR data of **5** were comparable to those of **4**. Observed in the ^1^H-NMR spectra was the absence of proton signal of an oxymethine at H-11 (δ_H_ 4.28, t, *J* = 7.0 Hz, 1H), with the appearance of an additional oxygenated methylene proton resonances at δ_H_ 3.58 (d, *J* = 10.2 Hz, 1H) and 3.80 (d, *J* =10.2 Hz, 1H) in the ^1^H-NMR spectra of **5**. Correspondingly, an oxymethine carbon at δ_C_ 65.9 (C-11) was absent with the presence of an oxygenated secondary carbon at δ_C_ 72.5 in the ^13^C-NMR spectra of **5**. The HMBC correlations from H-23a (δ_H_ 3.58, d, *J* = 10.2 Hz, 1H) and H-23b (δ_H_ 3.80, d, *J* = 10.2 Hz, 1H) to C-3 (δ_C_ 220.9), C-4 (δ_C_ 54.3) and C-5 (δ_C_ 54.2) indicated that a hydroxyl group was attached to C-23, which was also confirmed by the key ROESY correlations (Figure 3) of H-23/H-5, as well as the downfield shifted carbon resonance of C-4, as compared with those of compound **4**. Analysis of the ROESY spectrum suggested the relative configuration of the remainder of the molecule of **5** was identical with that of **4**. Compound **5** (davinvolunone C) was therefore determined as 6α,23,29-trihydroxy-d-friedoolean-14-en-3,16,21-trione.

The novel compounds, namely davinvolunols A–B (**1**–**2**) and davinvolunones A–C (**3**–**5**), are all taraxerene-type triterpenoids with a d-friedoolean-14-en skeleton. To the best of our knowledge, compounds **1**–**3**, as well as previously reported two ursane triterpenes, davinvolunic acid A and B from the same plant [13], are the first reported 2-nor pentacyclic triterpenoids with 29 carbons and a five-membered A-ring. The results provided important, evolutionary and chemotaxonomic knowledge of monotypic genus *Davidia*.

The isolated triterpenoids were evaluated for cytotoxic activities against SGC-7901 human gastric cancer cells, MCF-7 human breast cancer cells and BEL-7404 human hepatoma cells. The cytotoxicities were summarized in Table 1. Among the pure triterpenoids isolated from *Davidia involucrata*, compounds **3**–**5**, **7** and **9** showed moderate cytotoxic activities against all three cell lines. Moreover, davinvolunone B (**4**) exhibited the most potent cytotoxicities towards SGC-7901, MCF-7, and BEL-7404 cancer cells with IC_50_ value of 30.57 ± 1.63, 41.34 ± 1.24, and 37.29 ± 1.64 μM, respectively.

**Table 1:** Cytotoxicities of compounds **1**–**7** and **9** against three human tumor cell lines (IC_50_, μM).

Compounds	Cell Lines		
	SGC-7901	MCF-7	BEL-7404
**1**	>100	>100	>100
**2**	>100	>100	>100
**3**	58.23 ± 2.36	65.35 ± 3.12	70.26 ± 4.21
**4**	30.57 ± 1.63	41.34 ± 1.24	37.29 ± 1.64
**5**	32.22 ± 1.22	42.54 ± 1.67	39.26 ± 1.14
**6**	>100	>100	>100
**7**	35.47 ± 1.55	55.24 ± 2.28	53.82 ± 2.11
**9**	37.65 ± 1.85	58.93 ± 2.58	54.77 ± 2.05
Doxorubicin	0.19 ± 0.032	0.08 ± 0.007	0.12 ± 0.011

## 3. Experimental Section

### 3.1. General

Optical rotations were measured with a Jasco DIP-180 digital polarimeter. IR spectra were recorded with a Perkin-Elmer 1750 FT-IR spectrometer in KBr discs. High-resolution mass spectra were recorded on an IonSpec 4.7 Tesla FTMS instrument. The NMR spectra were obtained by using a Bruker AV-400 or a DRX-500 spectrometer. Semipreparative HPLC was performed with an Elite P230 pump equipped with a Schambeck SFD GmbH RI2000 detector and a YMC-Pack SIL column (250 × 10 mm, 5 µm). Sephadex LH-20 (25–100 µm, Pharmacia Fine Chemicals, Uppsala, Sweden), Silica gel (200–300 mesh) and Silica gel H (Qingdao Oceanic Chemical Co., Qingdao, China) were used for column chromatography. Thin-layer chromatography was performed on TLC plates (H, Qingdao Oceanic Chemical Co.), with compounds visualized by spraying with 5% (v/v) H_2_SO_4_ in alcohol solution, followed by heating.

### 3.2. Plant Material

The branch barks of *D. involucrata* were collected from Shennongjia Forest Region of Hubei province, P. R. China in 2002. The plant was authenticated by Prof. Y. P. Yang. A voucher specimen (No. 12245) is deposited in the Herbarium of Kunming Institute of Botany, Chinese Academy of Sciences, Kunming, China.

### 3.3. Extraction and Isolation

The air-dried branch barks of the plant (10 kg) were extracted with MeOH (2 × 10 L) at room temperature to give 600 g of crude extract, which was solubilized in water (1 L) and then filtered. The water-insoluble fraction (175 g) was separated on a silica gel column (200–300 mesh, 80 × 5 cm, i.d.) that was eluted with a gradient of Petroleum ether/Acetone (from 10:1 to 10:5, v/v) to afford seven fractions 1–7.

Fraction 5 (6 g) was subjected to Sephadex LH-20 column (120 × 3 cm, i.d.) eluting with CHCl_3_/MeOH (1:1, v/v) to afford five fractions 5-1–5-5. Fraction 5-2 was subjected to chromatography over a silica gel column (silica gel H, 25 × 2.5 cm, i.d.) with a gradient of Petroleum ether/CHCl_3_/EtOAc (10:6:2, v/v) to yield **1** (11.0 mg). Fraction 5-3 was purified by chromatography over a silica gel column (silica gel H, 25 × 2.5 cm, i.d.) eluting with a gradient of Petroleum ether/Acetone/MeOH (10/20/2, v/v) to afford **2** (15.2 mg), **3** (16.6 mg) and **6** (21.4 mg).

And fraction 7 (8 g) was subjected to Sephadex LH-20 column (120 × 3 cm, i.d.) chromatography eluting with MeOH to afford four fractions 7-1–7-4. Fraction 7-4 was then purified by semi-preparative HPLC with a gradient of CHCl_3_–MeOH (100:4, v/v) to give **4** (7.2 mg) and **5** (6.3 mg).

### 3.4. Spectral Data

*Davinvolunol A* (**1**): white amorphous powder; 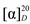 = +99.0 (c 0.15, CHCl_3_); IR (KBr) ν_max_: 3560, 2950, 1640, 1470 cm^−1^; HR-TOF-MS *m/z* = 435.3596 [M + Na]^+^ (calcd for C_29_H_48_ONa, 435.3603); ^1^H-NMR (500 MHz, CDCl_3_ and CD_3_OD) and ^13^C-NMR (125 MHz, CDCl_3_ and CD_3_OD) data (Table 2 and Table 3).

**Table 2:** ^1^H-NMR Data of compounds **1**–**5**.

Position	1^a^ [δ_H_ (*J* in Hz)]	2^b^ [δ_H_ (*J* in Hz)]	3^c^ [δ_H_ (*J* in Hz)]	4^d^ [δ_H_ (*J* in Hz)]	5^d^ [δ_H_ (*J* in Hz)]
1	0.80, m	0.84, m	1.60, d (14.3)	2.05, m	1.80, m
	1.53, m	1.51, m	1.79, d (14.3)	2.12, m	1.85, m
2	–	–	–	2.30, m	2.32, m
				2.75, ddd (16.3, 11.2, 4.9)	2.59, ddd (16.3, 11.2, 6.4)
3	2.01, m	3.18, dd (9.8, 6.8)	–	–	–
	2.25, m				
4	–	–	–	–	–
5	1.00, m	0.98, m	1.61, d (10.9)	1.78, d (11.1)	2.03, d (11.0)
6	1.23, m	1.21, m	3.91, ddd (15.0, 10.9, 4.1)	3.98, ddd (15.2, 11.1, 4.2)	3.98, ddd (15.2, 11.2, 4.0)
	1.83, d (13.0)	1.80, d (13.0)			
7	1.35, m	1.27, m	1.38, t (10.9)	1.49, t (11.1)	1.50, t (11.2)
	2.03 dt-like (13.0)	2.01 dt-like (13.0)	2.28, dd (10.9, 4.1)	2.30, dd (11.1, 4.2)	2.28, dd (11.2, 4.0)
8	–	–	–	–	–
9	1.49, m	1.51, m	1.52, m	1.60, d (7.0)	1.70, m
10	–	–	–	–	–
11	1.51, m	1.50, m	1.60, m	4.28, t (7.0)	1.85, m
	1.65 m	1.60, m	1.72, m		1.92, m
12	1.48, m	1.46, m	1.60, m	1.98, d (14.5)	1.80, m
	1.60, m	1.53, m	1.75, m	2.12, d (14.5)	1.92, m
13	–	–	–	–	–
14	–	–	–	–	–
15	5.51, dd (8.2, 3.1)	5.47, dd (8.0, 3.1)	5.60, dd (8.1, 2.9)	5.96, br. s	5.94, br. s
16	1.68, dd (15.2, 3.1)	1.66, dd (15.2, 3.1)	1.79, dd (14.5, 8.1)	–	–
	2.13, dd (15.2, 8.2)	2.09, dd (15.2, 8.0)	2.05, dd (14.5, 2.9)		
17	–	–	–	–	–
18	0.57, dd (13.5, 3.8)	0.60, dd (13.5, 3.8)	1.22, dd (13.5, 5.1)	1.88, dd (13.5, 5.2)	1.92, dd (13.5, 5.1)
19	0.91, dd (13.5, 3.8)	0.96, dd (13.5, 3.8)	1.44, dd (13.5, 5.1)	1.63, dd (13.5, 5.2)	1.65, dd (13.5, 5.1)
	1.37, t (13.5)	1.32, t (13.5)	1.88, t (13.5)	2.54, t (13.5)	2.49, t (13.5)
20	–	–	–	–	–
21	1.53, m	1.46, m	–	–	–
	1.65, m	1.60, m			
22	1.24, m	1.20, m	1.87, d (12.8)	2.51, d (13.8)	2.49, d (13.7)
	2.05, m	2.03, m	2.63, d (12.8)	2.62, d (13.8)	2.61, d (13.7)
23	1.22, s	1.05, s	1.29, s	1.35, s	3.58, d (10.2)
					3.80, d (10.2)
24	0.94, s	0.91, s	1.32, s	1.36, s	1.27, s
25	0.87, s	0.84, s	0.82, s	0.98, s	1.13, s
26	1.08, s	1.02, s	1.11, s	1.26, s	1.30, s
27	1.07, s	1.01, s	1.02, s	1.22, s	0.96, s
28	3.10 (d, 10.9)	3.06 (d, 10.9)	0.78, s	1.07, s	1.06, s
	3.23, (d, 10.9)	3.18, (d, 10.9)			
29	1.05, s	1.02, s	1.06, s	3.26, d (10.3)	3.24, d (10.4)
				3.71, d (10.3)	3.69, d (10.4)
30	0.94, s	0.90, s	1.05, s	1.06, s	1.05, s

^a^ Recorded in CDCl_3_ and CD_3_OD (10:1) in 500 MHz; ^b^ Recorded in CDCl_3_ and CD_3_OD (10:1) in 400 MHz; ^c^ Recorded in CDCl_3_ in 400 MH; ^d^ Recorded in CD_3_OD in 400 MHz.

**Table 3:** ^13^C-NMR Data of compounds **1**–**5**.

Position	1^a^ (δ_c_ mult.)	2^b^ (δ_c_ mult.)	3^c^ (δ_c_ mult.)	4^d^ (δ_c_ mult.)	5^e^ (δ_c_ mult.)
1	38.2 (CH_2_)	38.1 (CH_2_)	37.3 (CH_2_)	39.3 (CH_2_)	36.4 (CH_2_)
2	–	–	–	34.1 (CH_2_)	31.6 (CH_2_)
3	29.8 (CH_2_)	77.3 (CH)	219.7 (C)	222.5 (C)	220.9 (C)
4	47.5 (C)	47.7 (C)	47.0 (C)	49.2 (C)	54.3 (C)
5	55.7 (CH)	55.7 (CH)	58.8 (CH)	59.9 (CH)	54.2 (CH)
6	19.9 (CH_2_)	19.8 (CH_2_)	67.3 (CH)	67.5 (CH)	67.2 (CH)
7	40.6 (CH_2_)	40.5 (CH_2_)	50.4 (CH_2_)	50.2 (CH_2_)	50.3 (CH_2_)
8	38.9 (C)	38.9 (C)	39.7 (C)	41.2 (C)	42.5 (C)
9	48.5 (CH)	48.5 (CH)	47.0 (CH)	55.9 (CH)	47.2 (CH)
10	40.3 (C)	40.3 (C)	38.5 (C)	40.7 (C)	40.0 (C)
11	17.3 (CH_2_)	17.2 (CH_2_)	16.8 (CH_2_)	65.9 (CH)	17.7 (CH_2_)
12	30.6 (CH_2_)	30.5 (CH_2_)	33.2 (CH_2_)	43.3 (CH_2_)	37.4 (CH_2_)
13	37.6 (C)	37.6 (C)	37.3 (C)	38.9 (C)	39.3 (C)
14	158.5 (C)	158.3 (C)	157.3 (C)	177.9 (C)	178.3 (C)
15	116.0 (CH)	116.1 (CH)	116.5 (CH)	118.8 (CH)	119.4 (CH)
16	32.6 (CH_2_)	32.5 (CH_2_)	37.5 (CH_2_)	206.3 (C)	206.3 (C)
17	37.9 (C)	37.9 (C)	37.3 (C)	47.1 (C)	47.2 (C)
18	44.8 (CH)	44.7 (CH)	48.8 (CH	46.4 (CH)	46.7 (CH)
19	35.7 (CH_2_)	35.7 (CH_2_)	37.3 (CH_2_	31.4 (CH_2_)	31.8 (CH_2_)
20	28.9 (C)	28.8 (C)	43.1 (C)	48.7 (C)	48.5 (C)
21	33.3 (CH_2_)	33.2 (CH_2_)	219.7 (C)	219.9 (C)	220.0 (C)
22	27.8 (CH_2_)	27.6 (CH_2_)	51.5 (CH_2_)	48.4 (CH_2_)	48.2 (CH_2_)
23	25.6 (CH_3_)	25.9 (CH_3_)	31.5 (CH_3_)	32.2 (CH_3_)	72.5 (CH_2_)
24	21.3 (CH_3_)	21.3 (CH_3_)	19.7 (CH_3_)	20.6 (CH_3_)	16.4 (CH_3_)
25	14.7 (CH_3_)	14.7 (CH_3_)	16.0 (CH_3_)	17.9 (CH_3_)	17.1 (CH_3_)
26	25.7 (CH_3_)	25.7 (CH_3_)	25.6 (CH_3_)	25.5 (CH_3_)	24.6 (CH_3_)
27	21.6 (CH_3_)	21.5 (CH_3_)	20.2 (CH_3_)	29.1 (CH_3_)	26.9 (CH_3_)
28	65.1 (CH_2_)	64.7 (CH_2_)	32.5 (CH_3_)	33.8 (CH_3_)	33.9 (CH_3_)
29	33.4 (CH_3_)	33.3 (CH_3_)	28.1 (CH_3_)	71.2 (CH_2_)	71.2 (CH_2_)
30	29.8 (CH_3_)	29.7 (CH_3_)	23.0 (CH_3_)	20.2 (CH_3_)	20.2 (CH_3_)

^a^ Recorded in CDCl_3_ and CD_3_OD (10:1) in 125 MHz; ^b^ Recorded in CDCl_3_ and CD_3_OD (10:1) in 100 MHz; ^c^ Recorded in CDCl_3_ in 100 MHz; ^d^ Recorded in CD_3_OD in 100 MH; ^e^ Recorded in CD_3_OD in 125 MHz.

*Davinvolunol B* (**2**): white amorphous powder; 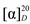 = +104.5 (c 0.17, CHCl_3_); IR (KBr) ν_max_: 3500, 2920, 1640, 1470 cm^−1^; HR-TOF-MS *m/z* = 451.3546 [M + Na]^+^ (calcd for C_29_H_48_O_2_Na, 451.3552); ^1^H-NMR (400 MHz, CDCl_3_ and CD_3_OD) and ^13^C-NMR (100 MHz, CDCl_3_ and CD_3_OD) data (Table 2 and Table 3).

*Davinvolunone A* (**3**): white amorphous powder; 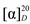 = +116.8 (c 0.16, CHCl_3_); IR (KBr) ν_max_: 3437, 2930, 1726, 1635, 1456 cm^−1^; HR-TOF-MS *m/z* 463.3180 [M + Na]^+^ (calcd for C_29_H_44_O_3_Na, 463.3188); ^1^H-NMR (400 MHz, CDCl_3_) and ^13^C-NMR (100 MHz, CDCl_3_) data (Table 2 and Table 3).

*Davinvolunone B* (**4**): white amorphous powder; 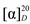 = +126.0 (c 0.12, CHCl_3_); IR (KBr) ν_max_: 3500, 2930, 1700, 1610, 1456 cm^−1^; HR-TOF-MS *m/z* 523.3030 [M + Na]^+^ (calcd for C_30_H_44_O_6_Na, 523.3036); ^1^H-NMR (400 MHz, CD_3_OD) and ^13^C-NMR (100 MHz, CD_3_OD) data (Table 2 and Table 3).

*Davinvolunone C* (**5**): white amorphous powder; 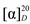 = +118.2 (*c* 0.20, CHCl_3_); IR (KBr) ν_max_: 3489, 2920, 1700, 1610, 1453 cm^−1^; HR-TOF-MS *m/z* 523.3030 [M + Na]^+^ (calcd for C_30_H_44_O_6_Na, 523.3036); ^1^H-NMR (400 MHz, CD_3_OD) and ^13^C-NMR (125 MHz, CD_3_OD) data (Table 2 and Table 3).

### 3.5. Cytotoxicity Assay

The *in vitro* cytotoxic activity was determined by the MTT colorimetric method as described previously in our paper [13]. Three tumor cell lines, SGC-7901 cells (human gastric adenocarcinoma), MCF-7 cells (human breast cancer) and BEL-7404 (human hepatocellular carcinoma), provided by Department of Hepatobiliary Surgery, Affiliated Union Hospital, Fujian Medical University, were cultured in RPMI-1640 medium supplemented with 10% fetal bovine serum and 100 IU·mL^−1^ of penicillin-streptomycin at 37 °C in humidified atmosphere with 5% CO_2_. For the cytotoxicity tests, cells in exponential growth stage were harvested from culture by trypsin digestion and centrifuging at 180*×* *g* for 3 min, and then resuspended in fresh medium at a cell density of 5 × 104 per mL. The cell suspension was dispensed into a 96-well microplate at 100 μL and incubated for 24 h, and then treated with compounds at various concentrations. After 48 h of treatment, 50 μL solution of 1 mg·mL^−1^ MTT was added to each well, and the cells were further incubated for 4 h. Finally, supernatants were removed and the formazan crystals were dissolved by adding 100 μL DMSO and the optical density was recorded at 570 nm. All drug doses were tested with doxorubicin as positive control in triplicate.

## 4. Conclusions

The phytochemical study of the methanol extract from the branch barks of *Davidia involucrata* has led to the isolation of five new taraxerene-type triterpenes, namely 2-nor-d-friedoolean-14-en-28-ol (**1**), 2-nor-d-friedoolean-14-en-3α,28-diol (**2**), 6α-hydroxy-2-nor-d-friedoolean-14-en-3,21-dione (**3**), 6α,11α,29-trihydroxy-d-friedoolean-14-en-3,16,21-trione (**4**), and 6α,23,29-trihydroxy-d-friedoolean-14-en-3,16,21-trione (**5**), in addition to five known compounds. This is the first report of the isolation of taraxerene-type triterpenes from the plant, and compounds **1**–**3** are rarely reported 2-nor pentacyclic triterpenoids with 29 carbons and a five-membered A-ring. Our studies suggested that pure triterpenoids isolated from the relic deciduous plant demonstrated moderate cytotoxic activities against the proliferation of SGC-7901, MCF-7, and BEL-7404 cell lines.

## Supplementary Materials

Supplementary materials can be accessed at: http://www.mdpi.com/1420-3049/19/11/17619/s1.

## Supplementary Files

Supplementary File 1
