# Supplementary material for: Five New Taraxerene-Type Triterpenes from the Branch Barks of Davidia involucrata
Source: Molecules. 2014 Oct 30;19(11):17619–31. doi: 10.3390/molecules191117619 (PMC6271488; doi:10.3390/molecules191117619)

Supplementary Material

Figure S1. ^{1}H-NMR spectrum (500 MHz) of Davinvolunol A (1) in $CDCl_3$ and CD_3OD (10:1).

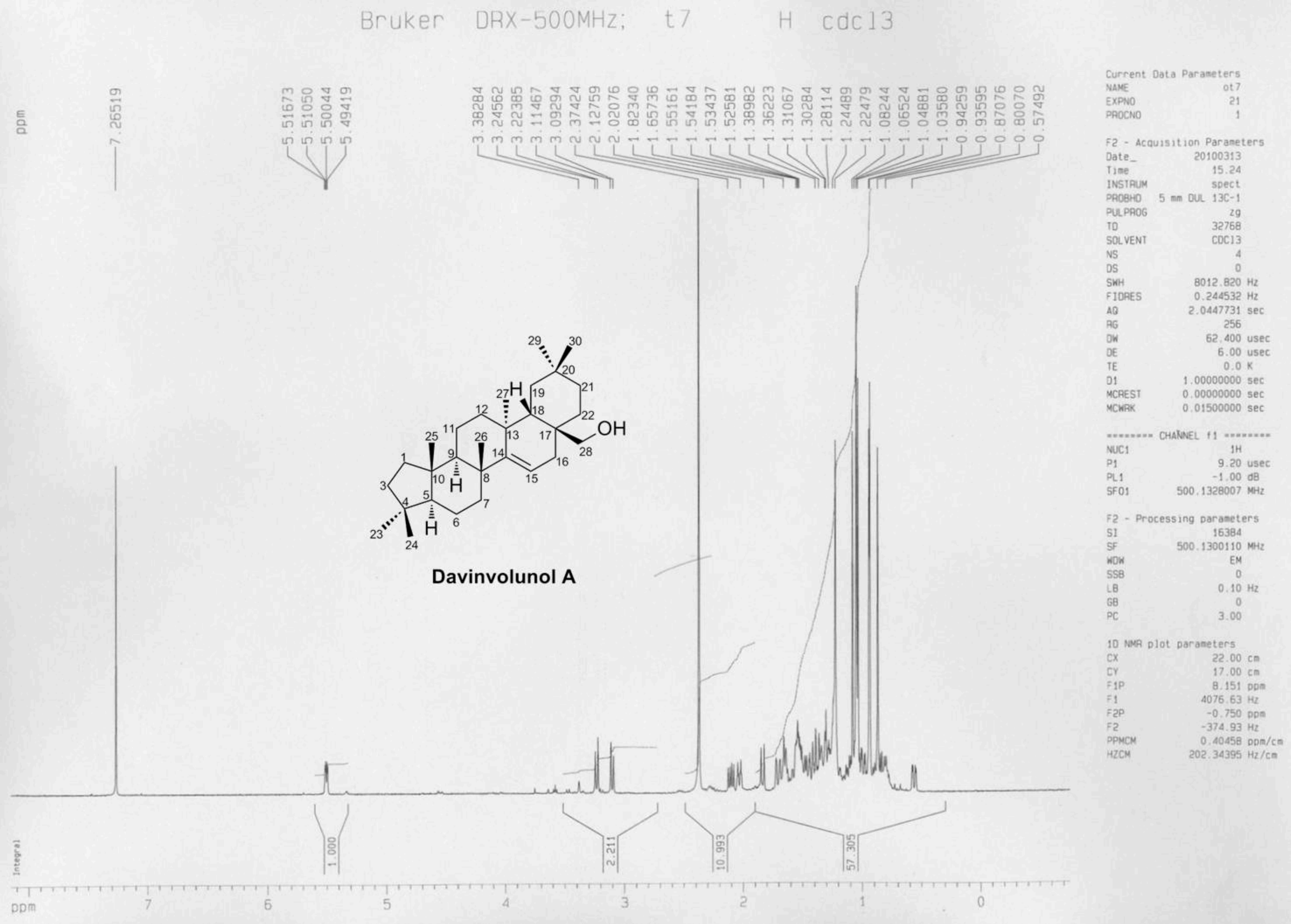

Figure S2. ^{13}C-NMR spectrum (125 MHz) of Davinvolunol A (1) in $CDCl_3$ and CD_3OD (10:1).

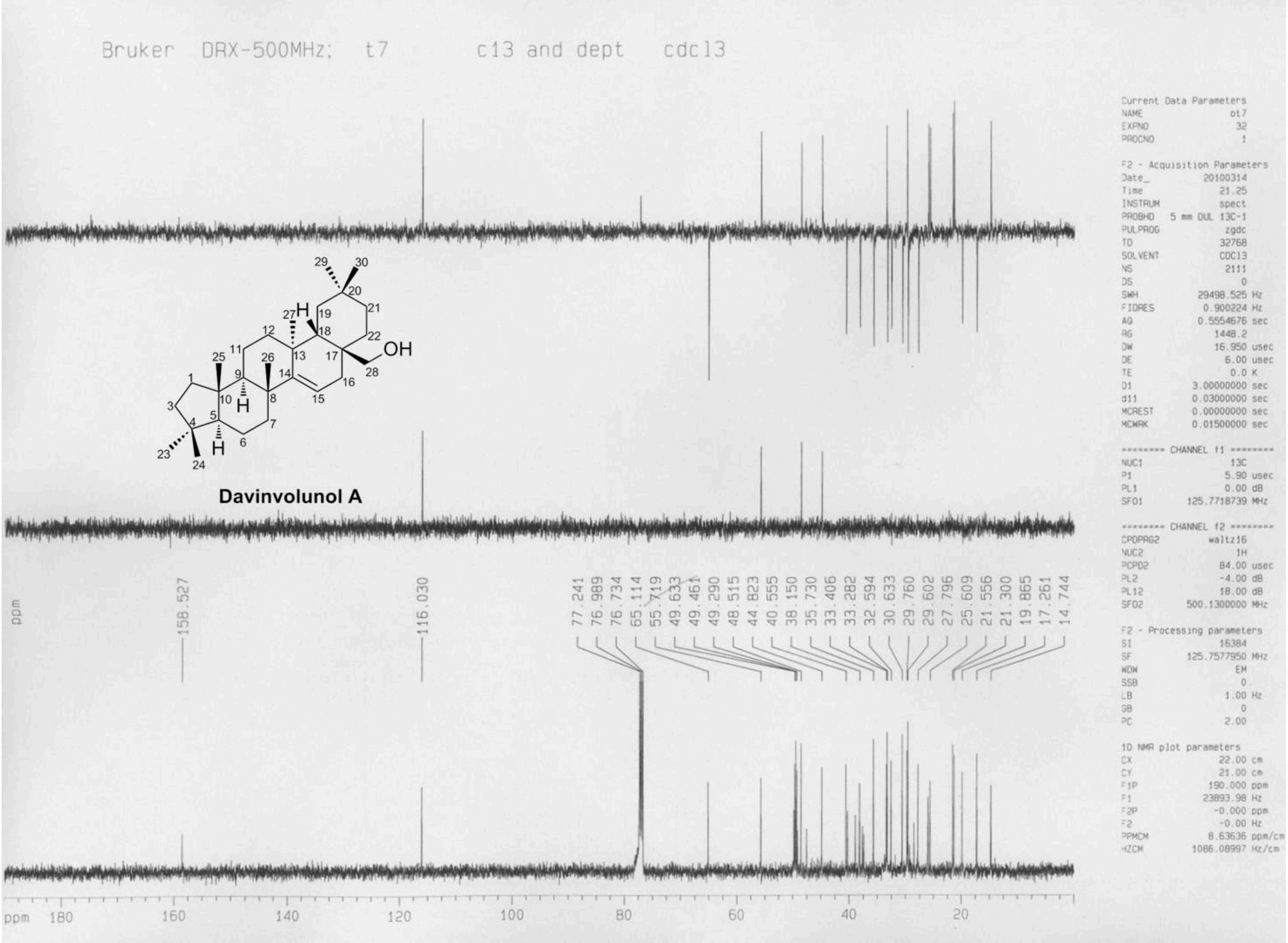

Figure S3. MALDI-TOF-MS spectrum of Davinvolunol A (1).

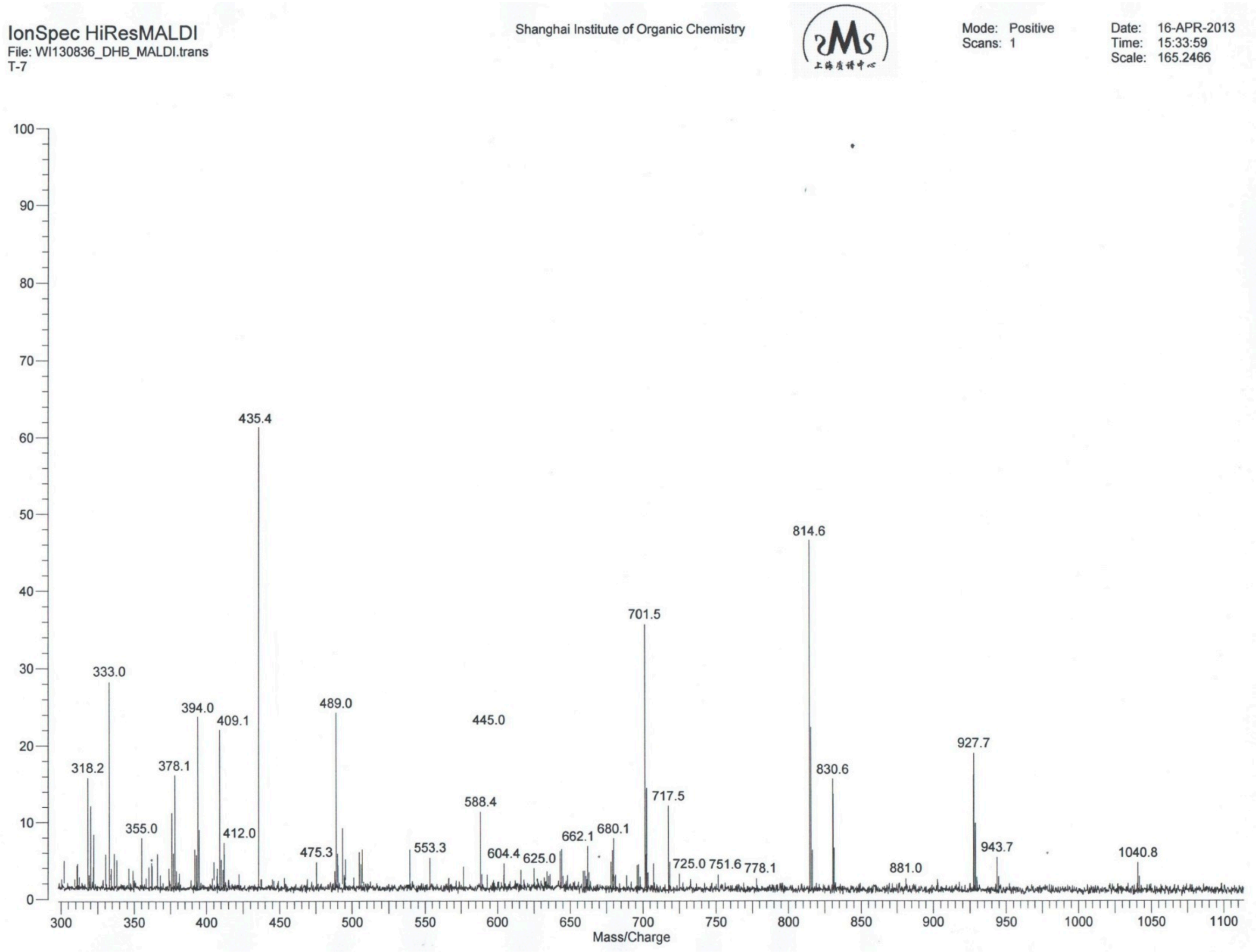

Figure S4. ^{1}H-NMR spectrum (400 MHz) of Davinvolunol B (2) in $CDCl_3$ and CD_3OD (10:1).

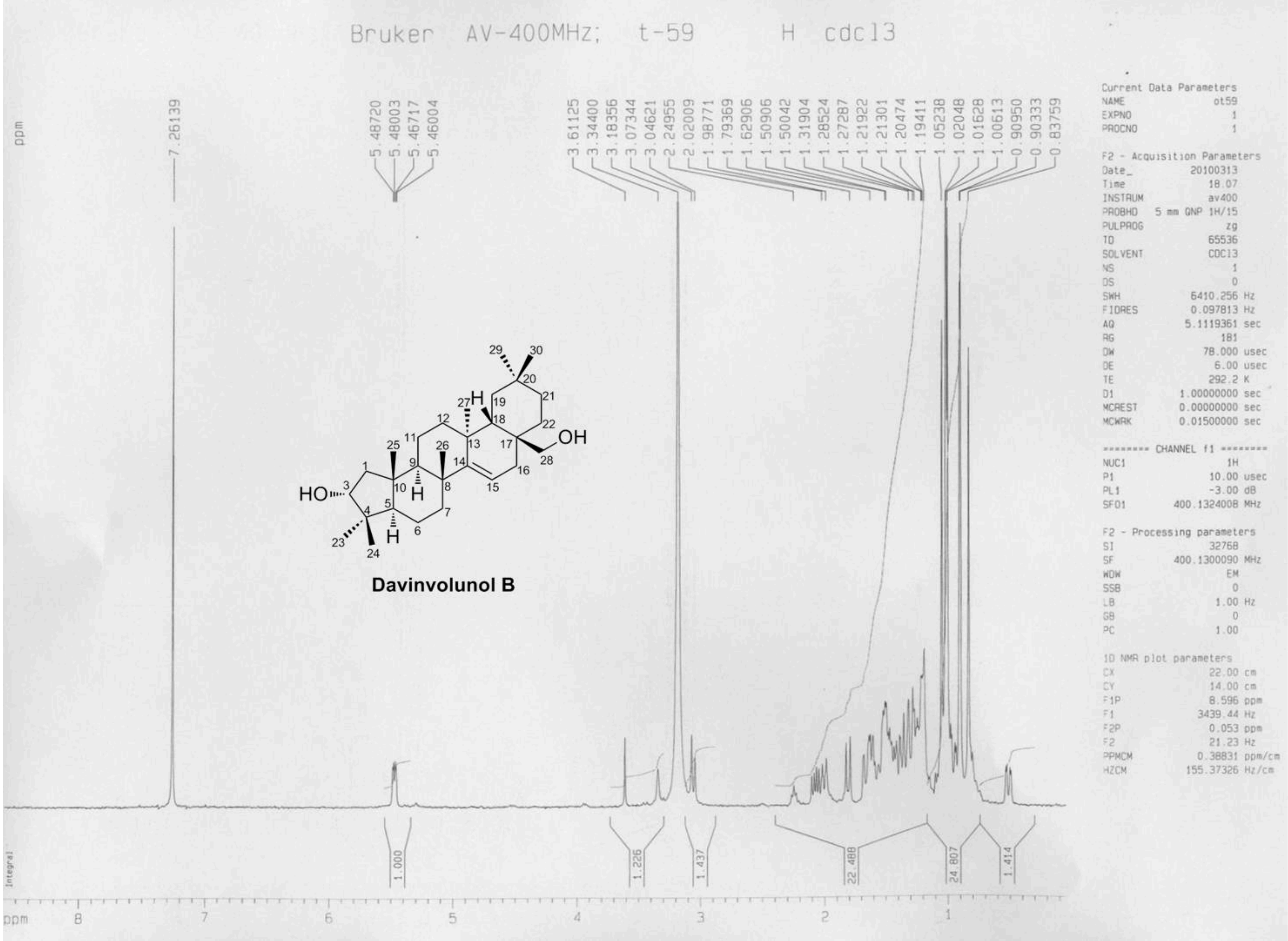

Figure S5. ^{13}C-NMR spectrum (100 MHz) of Davinvolunol B (2) in $CDCl_3$ and CD_3OD (10:1).

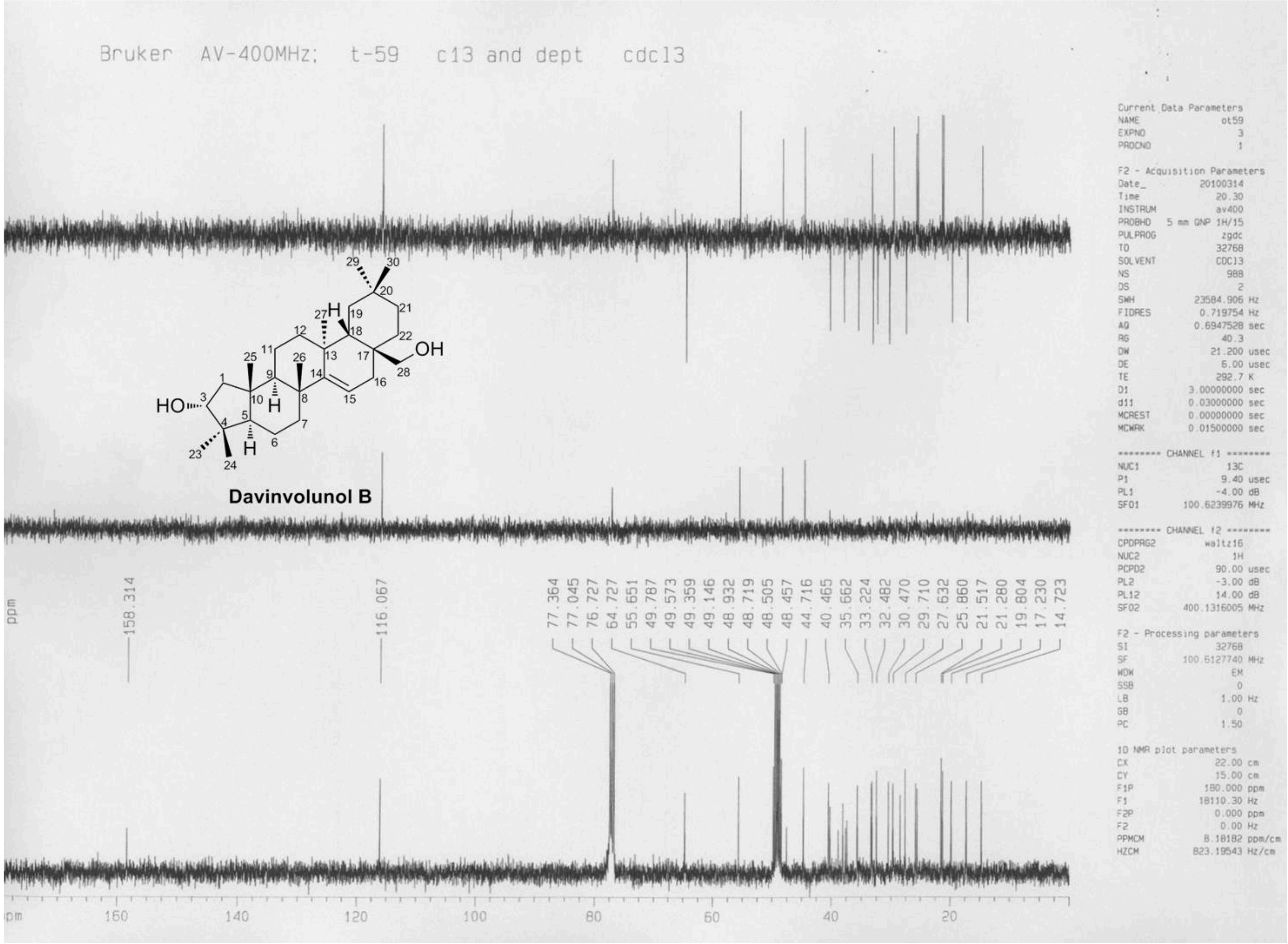

Figure S6. MALDI-TOF-MS spectrum of Davinvolunol B (2).

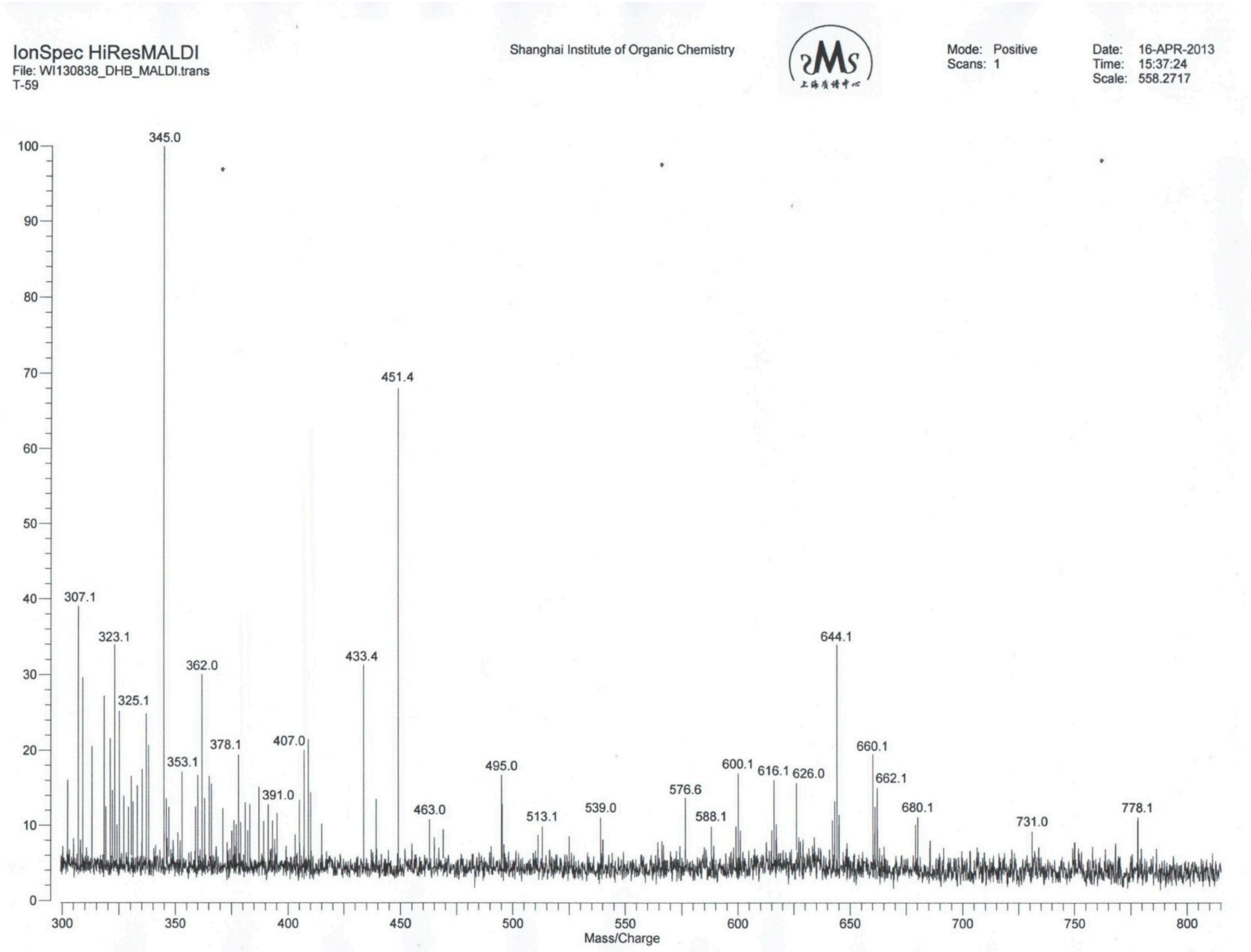

Figure S7. ^{1}H-NMR spectrum (400 MHz) of Davinvolunone A (3) in $CDCl_3$.

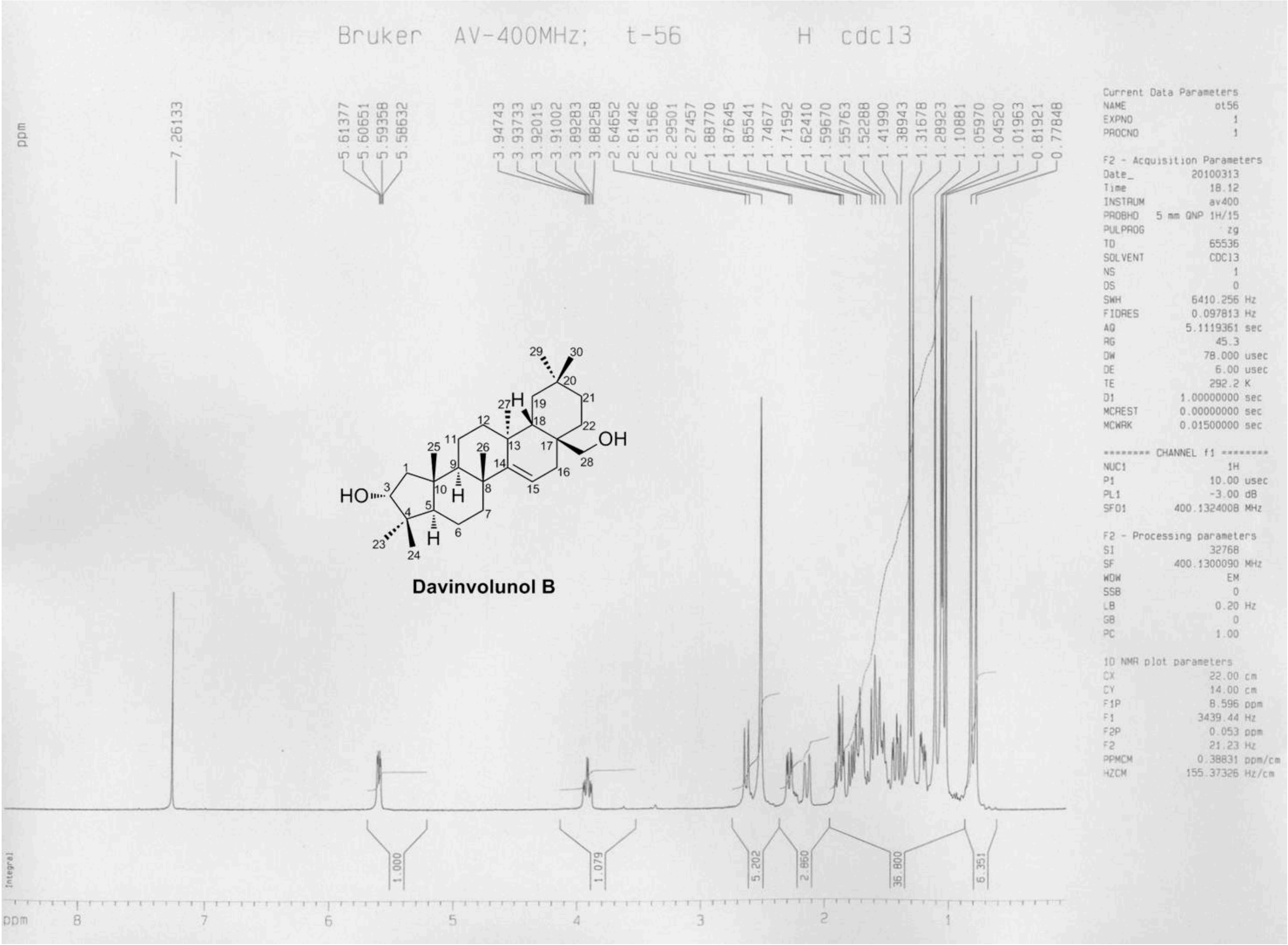

Figure S8. ^{13}C-NMR spectrum (100 MHz) of Davinvolunone A (3) in $CDCl_3$.

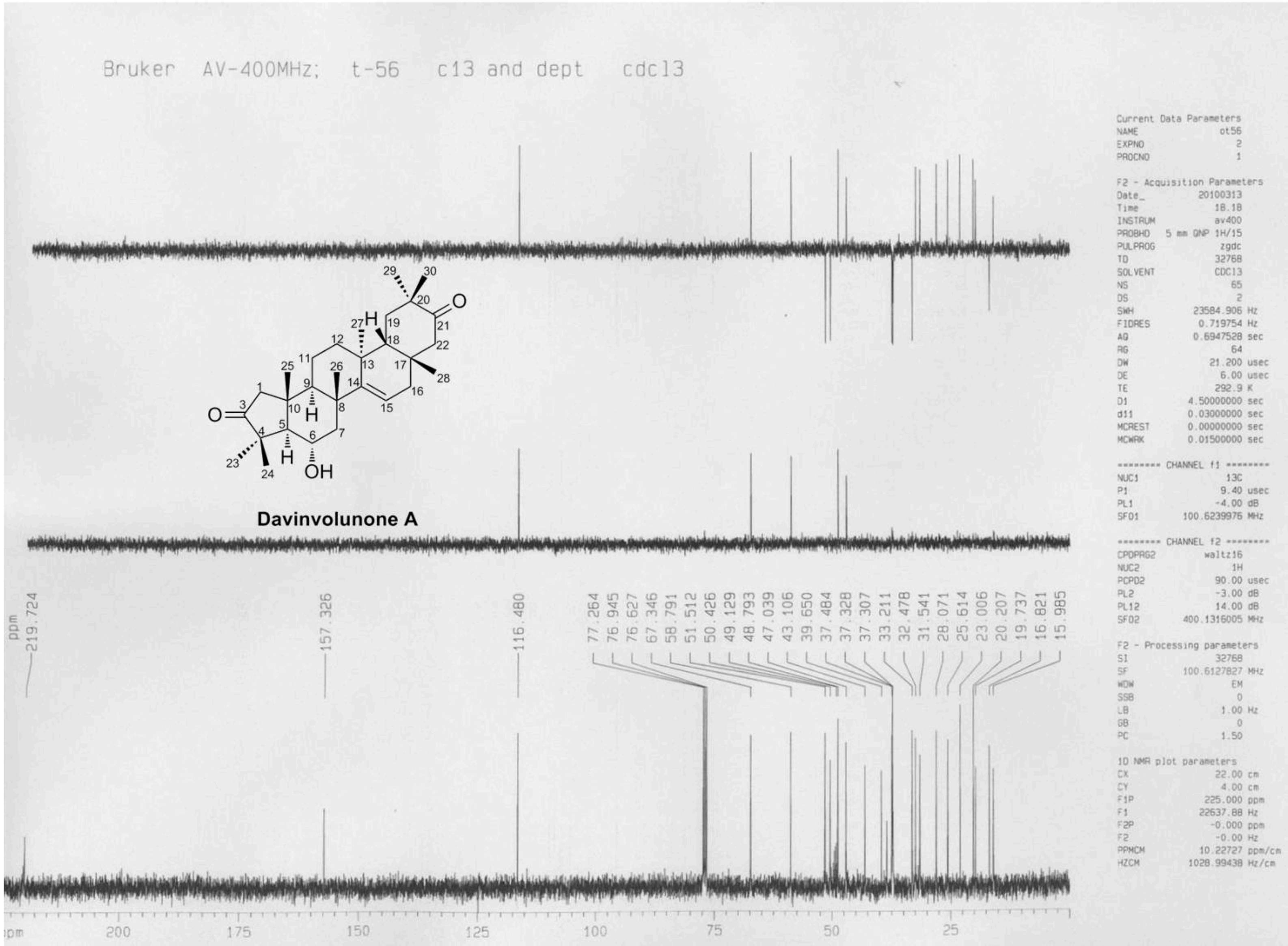

Figure S9. MALDI-TOF-MS spectrum of Davinvolunone A (3).

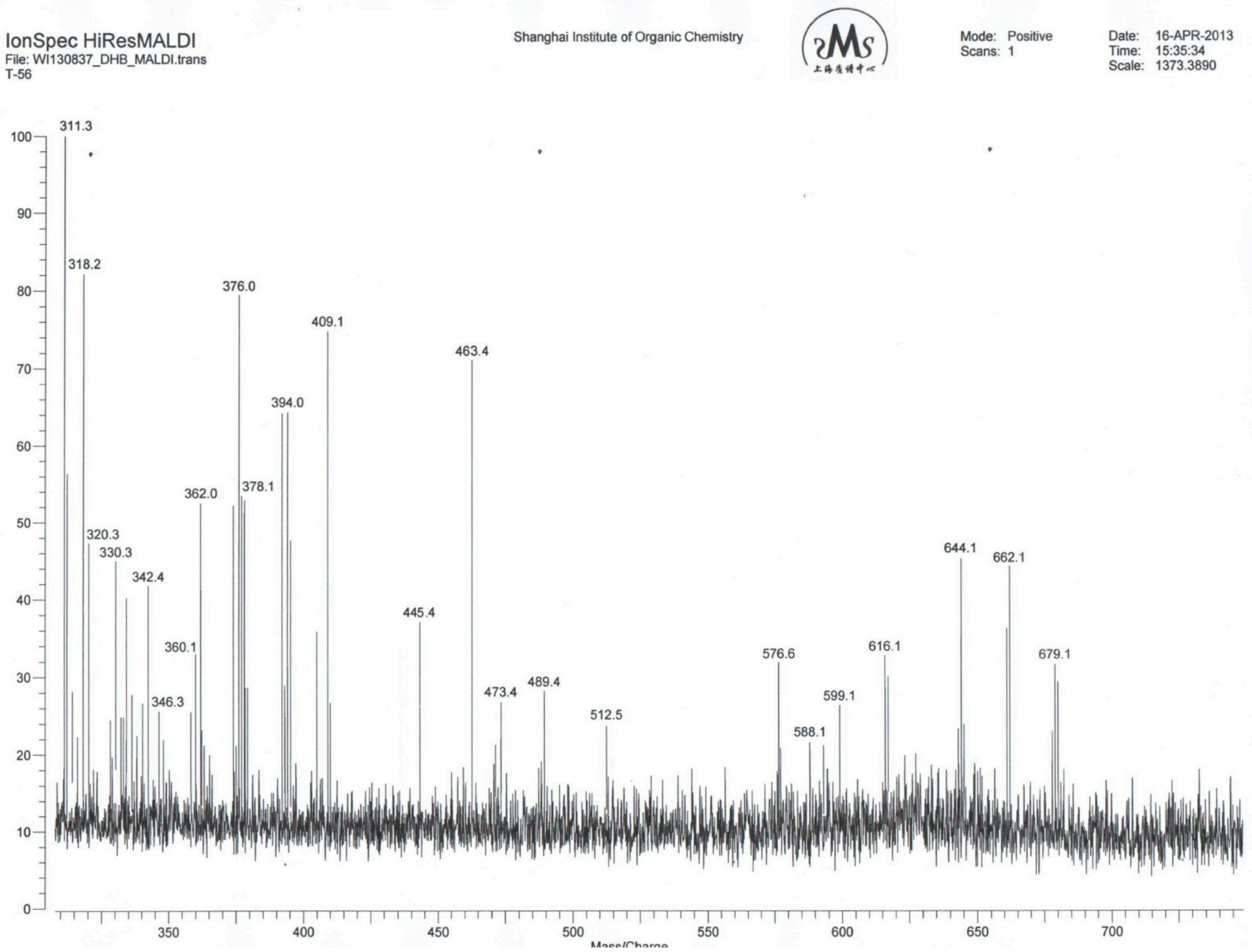

Figure S10. ^{1}H-NMR spectrum (400 MHz) of Davinvolunone B (4) in CD_3OD.

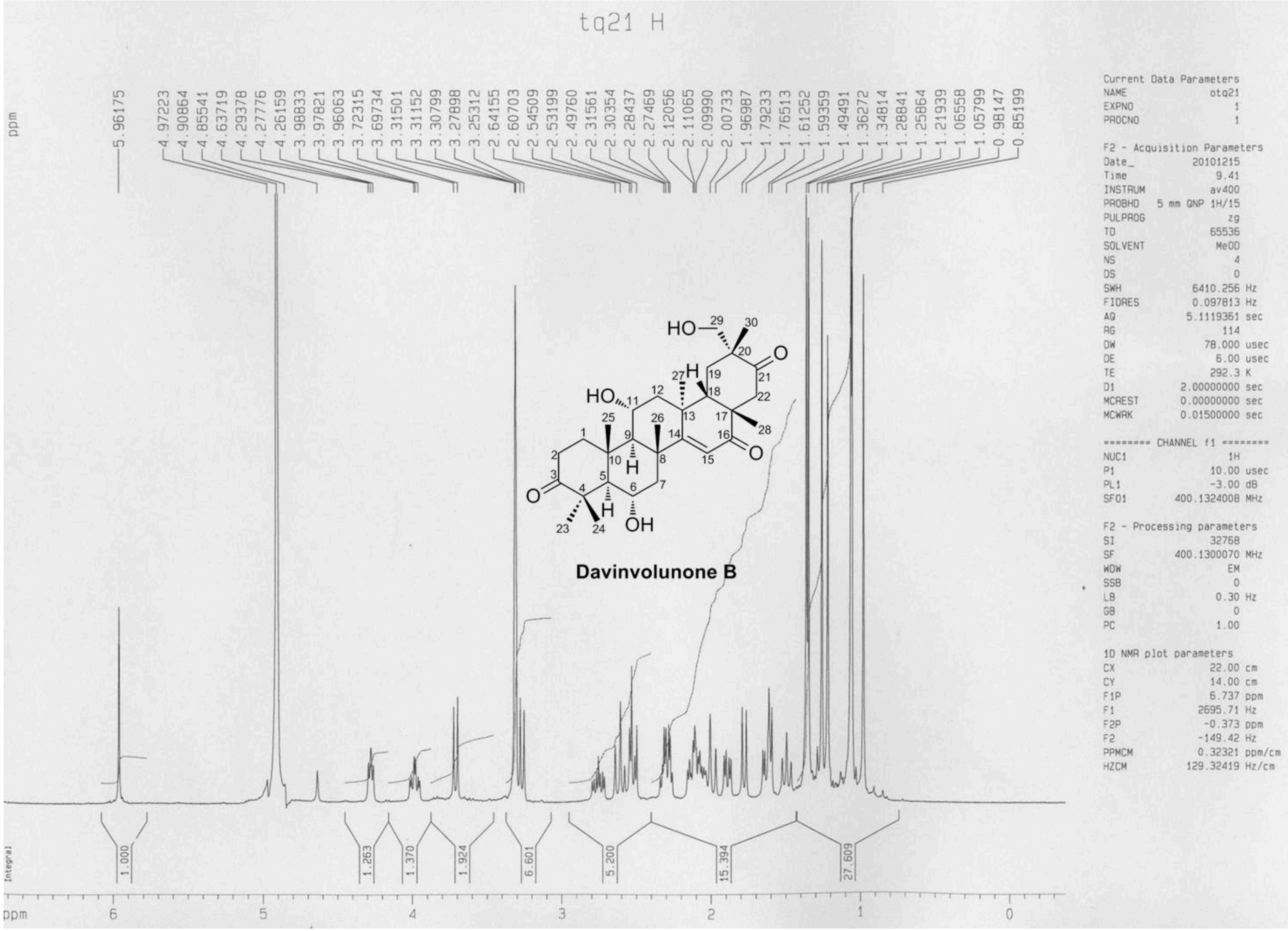

Figure S11. ^{13}C-NMR spectrum (100 MHz) of Davinvolunone B (4) in CD_3OD.

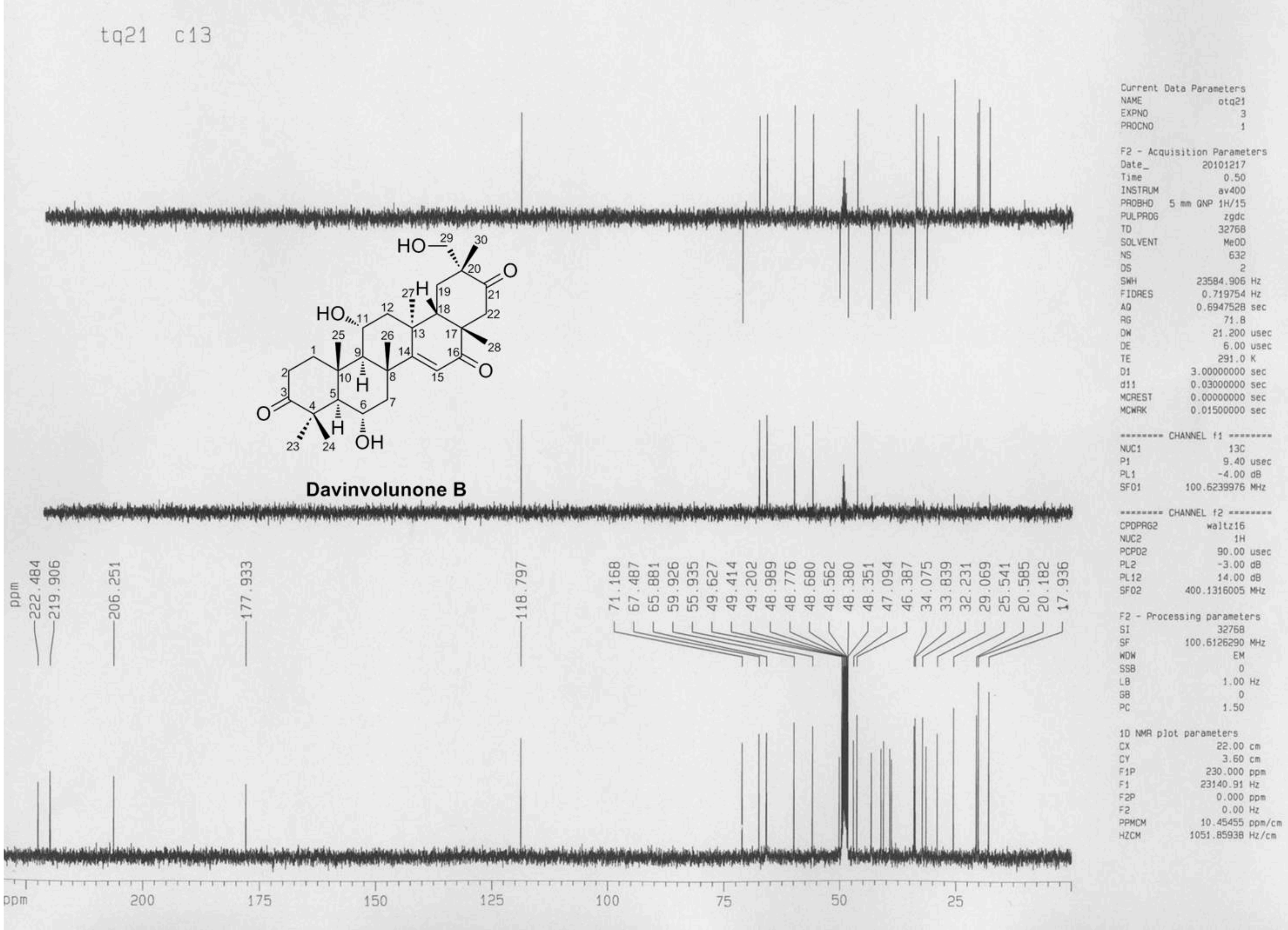

Figure S12. MALDI-TOF-MS spectrum of Davinvolunone B (4).

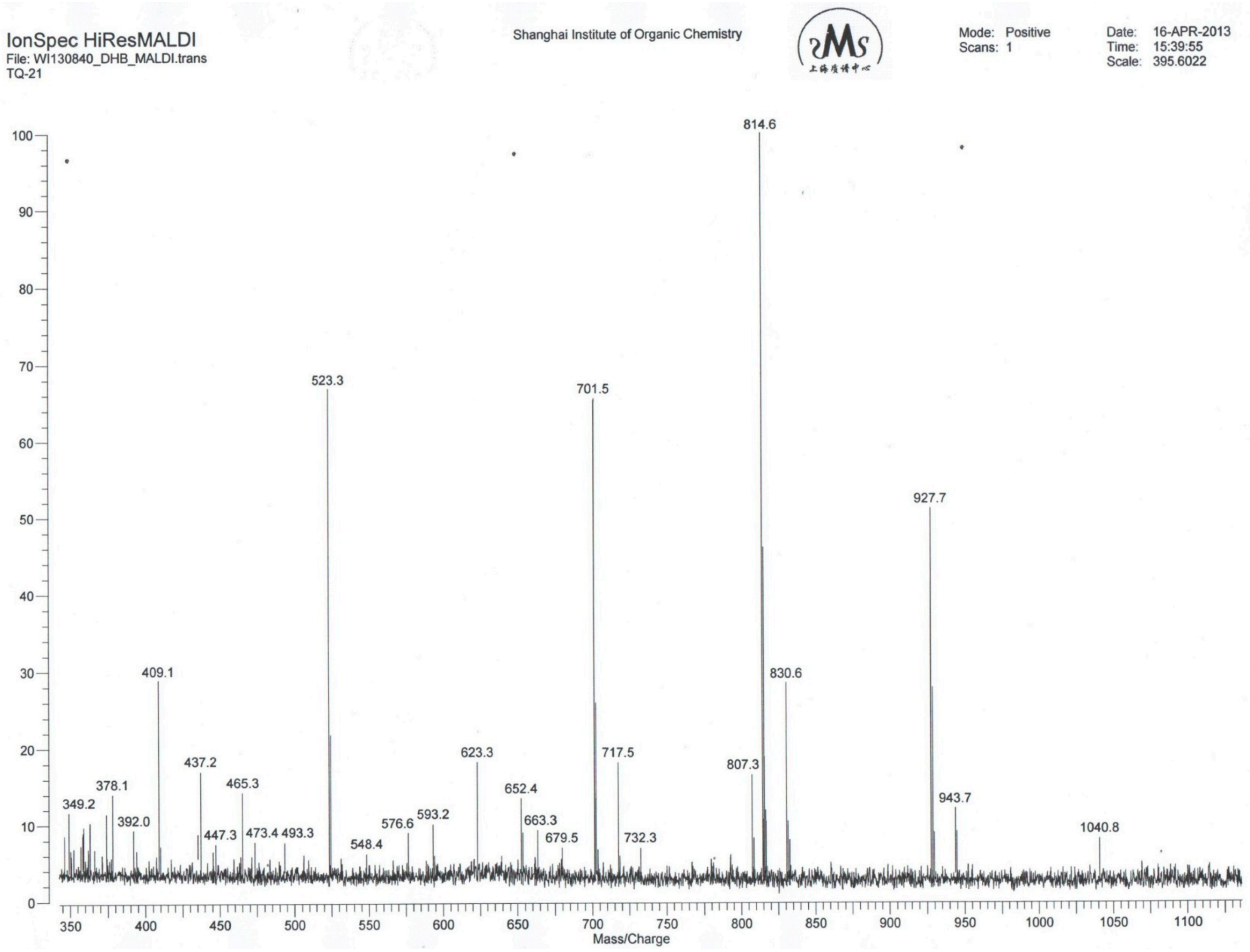

Figure S13. ^{1}H-NMR spectrum (400 MHz) of Davinvolunone C (5) in CD_3OD.

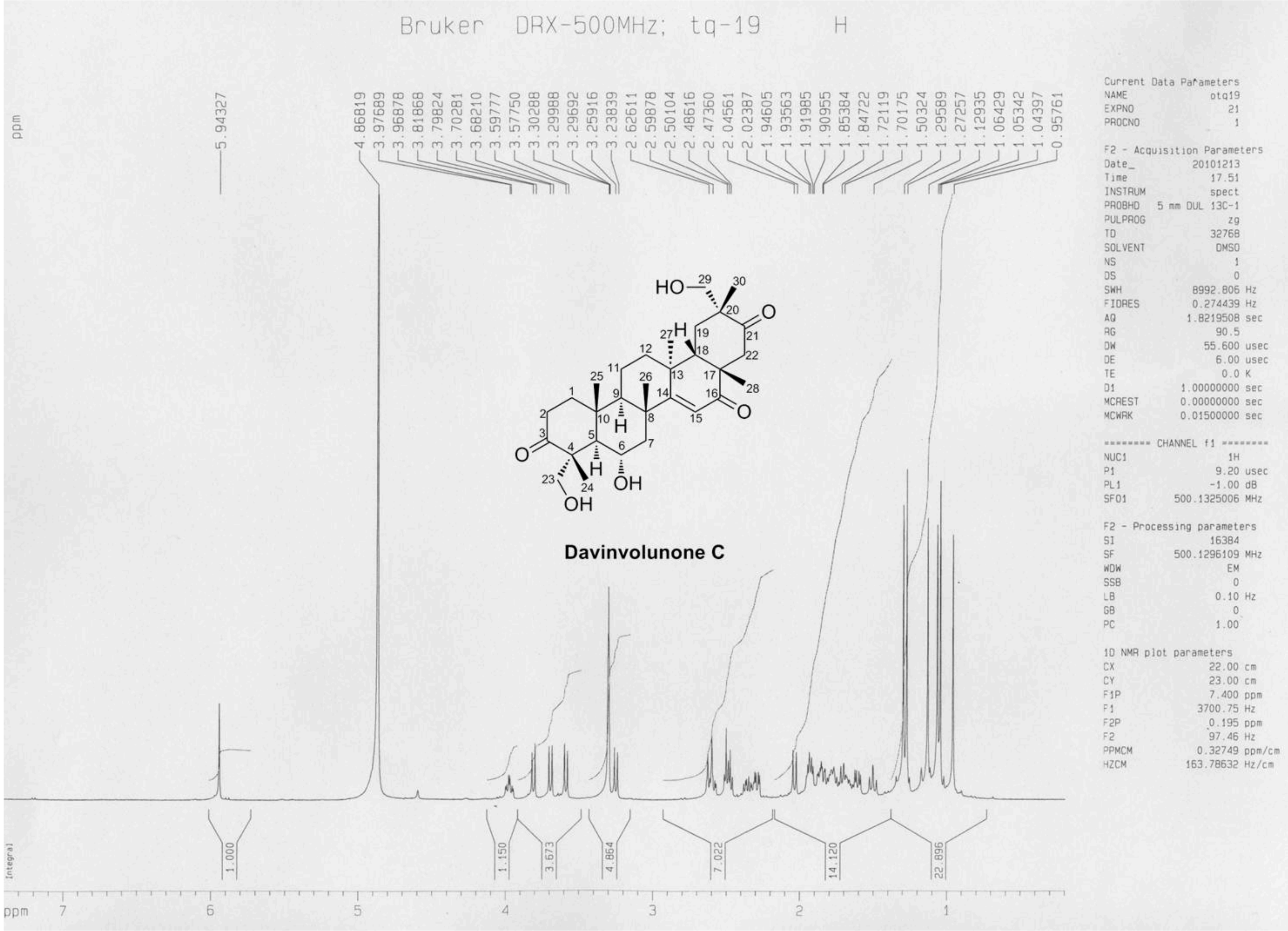

Figure S14. ^{13}C-NMR spectrum (125 MHz) of Davinvolunone C (5) in CD_3OD.

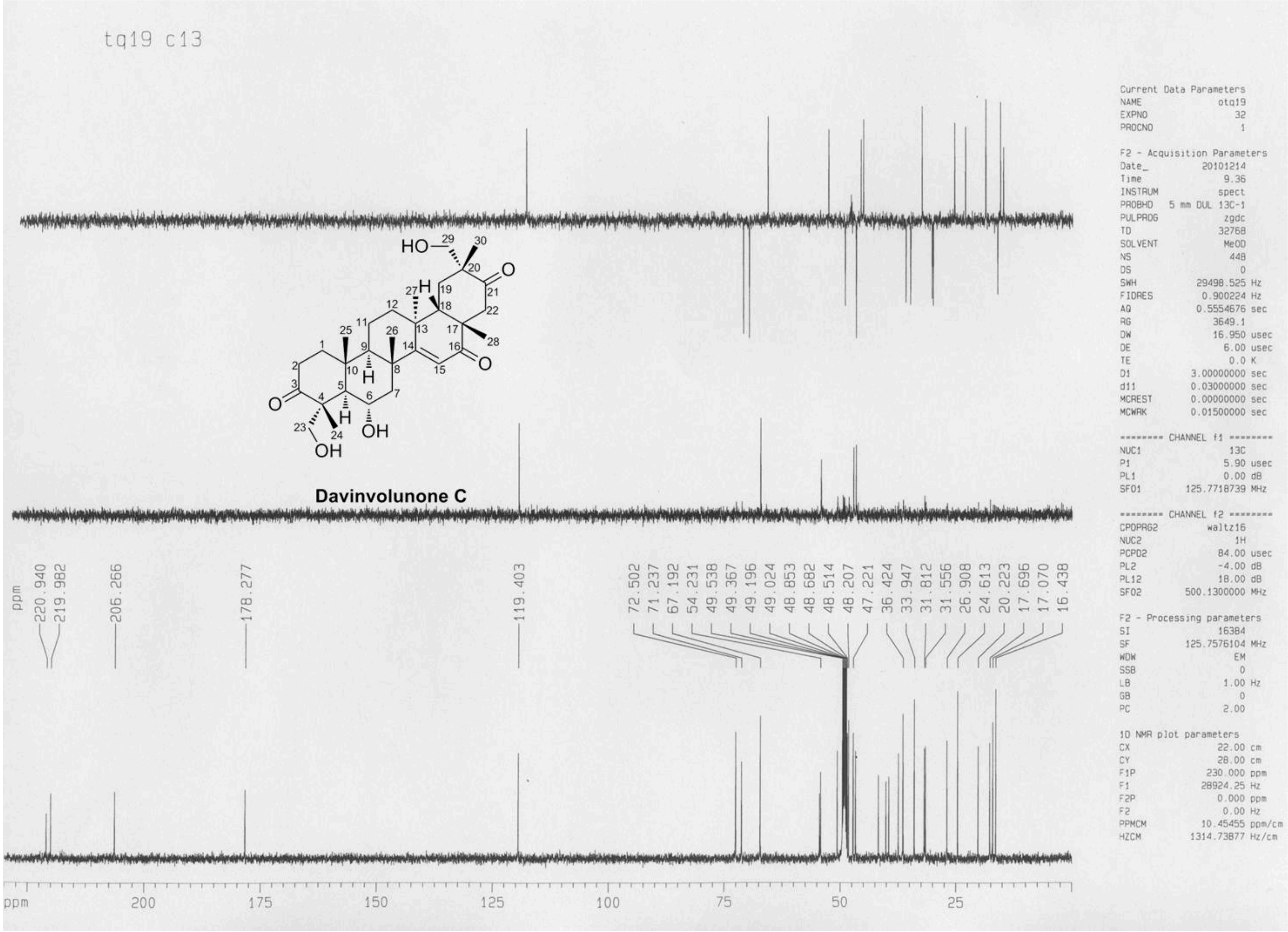

Figure S15. MALDI-TOF-MS spectrum of Davinvolunone C (5).

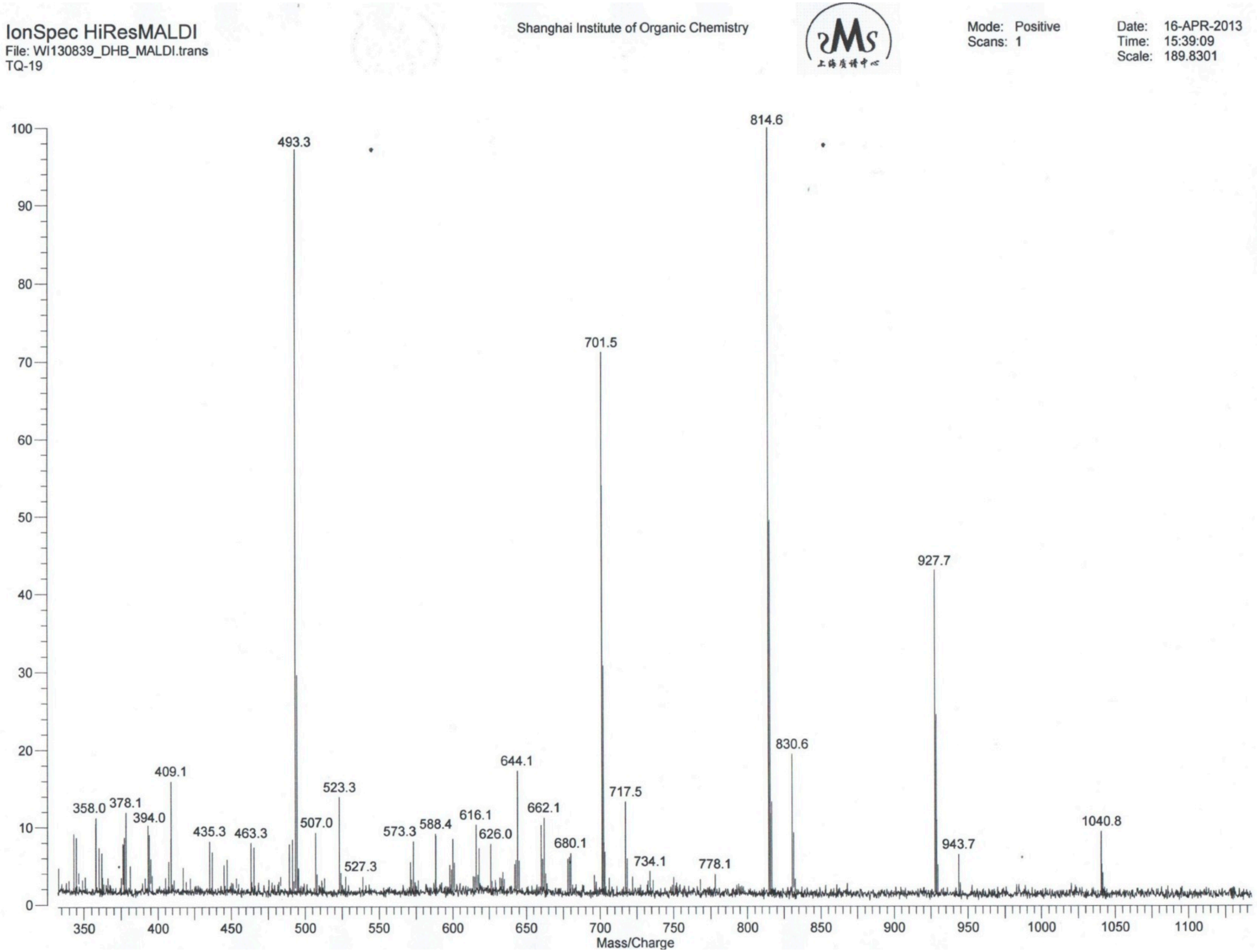

Supplement: Supplementary File 1 [file molecules-19-17619-s001.pdf]
